# Healthcare utilization and economics evaluation of paliperidone palmitate once-monthly in schizophrenia: a one-year, real-world, and retrospective mirror image study in China

**DOI:** 10.3389/fpsyt.2024.1415275

**Published:** 2024-09-04

**Authors:** Yixiang Zhou, Binbin Chen, Yinghua Huang

**Affiliations:** ^1^ Department of Pharmacy, Xiamen Xianyue Hospital, Xiamen, China; ^2^ Xianyue Hospital Affiliated with Xiamen Medical College, Xiamen, China; ^3^ Fujian Psychiatric Center, Xiamen, China; ^4^ Fujian Clinical Research Center for Mental Disorders, Xiamen, China; ^5^ Department of Clinical Pharmacy, Xiamen Xianyue Hospital, Xiamen, China

**Keywords:** schizophrenia, paliperidone palmitate, long-acting injection, medication compliance, health economic

## Abstract

**Background:**

Investigation and analysis of the changes in healthcare resources and burden of schizophrenia in the real world before and after switching from oral antipsychotics (OAPs) to paliperidone palmitate once-monthly (PP1M) could provide evidence to clinicians and patients for choosing treatment modality and data support for health policy optimization

**Methods:**

The first dosage of PP1M was set as mirror point, and patients with mirror point between January 2020 and June 2022 were recruited in the study. The differences in treatment patterns, healthcare resource utilization, and costs within one year before and after the mirror point were compared.

**Results:**

A total of 72 patients transitioning from OAPs to PP1M (mean age, 35.33 years; 43.06% female) were included in the study. Of the 72 patients, the application of PP1M resulted in a significant reduction in the hospitalization times, emergency room visits, and direct medical costs (P < 0.001), while the pharmacy cost and total cost increased by 222.57% (P < 0.001) and 16.35% (P < 0.001), respectively; PP1M accounted for 88.48% of the pharmacy cost. For patients with ≥1 hospitalization during the OAPs phase (n = 25), the number of hospitalizations, hospitalization days and hospitalization expenses decreased by more than 90% (P < 0.001). Total one-year expenses decreased by 37.67% (P < 0.001), and pharmacy expenses increased by 185.21% (P < 0.001). For patients with no hospitalizations during the OAPs phase (n = 47), emergency and outpatient visits decreased by 70% (P < 0.001) and 30.27% (P < 0.05), respectively, while the total cost increased by 117.56% (P < 0.001), and the pharmacy cost increased by 260.15% (P < 0.001) after initiation of PP1M treatment.

**Conclusion:**

After the transition to PP1M, the number of hospitalizations and outpatient and emergency department visits reduced, and healthcare resources were conserved. Switching to PP1M may be more economically beneficial for patients with prior hospitalizations while on OAP regimens. The high price of PP1M might be an obstacle to its widespread use.

## 1 Introduction

Schizophrenia (SZ), a serious mental disorder, is characterized by chronic persistence and easy recurrence. It is associated with positive, negative, emotional, and cognitive symptoms (1). A previously conducted systematic review and meta-analysis has demonstrated the recurrence rates of positive symptoms to be 28%, 43% and 54% after 1, 1.5–2, and 3 years of follow-up, respectively (2). Clinical outcomes indicated moderate or poor prognosis for more than 50% of patients following treatment (3). In addition, 54.5%–80% of patients failed to take medicine on time (4). Schizophrenia exerts a heavy care burden on families and care institutions, with 49% of caregivers being parents, and more than 50% of caregivers spending over 32 hours a week on care (5). Despite its very low incidence, this difficult-to-treat disease has accounted for 14% of the total global disease burden, which has been estimated to account for 0.02%–1.65% of the gross domestic product (6, 7).

Various factors contribute to the heavy economic burden associated with SZ; first, the disease leads to reduced physical function, resulting in the loss of productivity of patients and their families, including suspension from school and work, resulting in an elevated labor cost (8). Second, poor medication compliance directly contributes to poor clinical prognosis, high recurrence rate, and increased emergency visits and rehospitalizations, thereby contributing to increased direct medical expenses (9).

The advent of long-acting injectable (LAIs) antipsychotics, such as haloperidol decanoate injection, risperidone LAIs (RLAI), and PP1M, improved treatment compliance and are cost saving (10). The administration of PP1M also considerable improved compliance and resulted in reduced recurrence and readmissions (11, 12). The LAIs reduced the long-term burden of patients and their families and improved the quality of life of patients. LAIs applied in early-phase schizophrenia could reduce a significant and clinically meaningful 44% reduction in the incidence rate of first hospitalization, as well as a personally and economically important outcome and provide a lower risk of hospitalization or relapse of LAIs than OAPs. Thus, it might be especially meaningful for the patients at high risk of non-adherence by initiating LAIs (13, 14).

The advantages of PP1M, in comparison with OAPs or first-generation antipsychotic long-acting injectables (FGA-LAIs) have been confirmed by several studies (15, 16). Advantages included ensuring that clinicians are aware of noncompliance owing to missed or delayed injections, reducing the burden of oral tablets, and reducing the adverse consequences of planned or unplanned treatment intervals, which included the risk of relapse, hospitalization, and increased mortality (17). Initiation of PP1M required no oral complementation (18), and PP1M offered obvious pharmacoeconomic advantages compared with OAPs and other LAIs (19–21). Evidences of retrospective studies also demonstrated that significant decrease in relapse event rate, healthcare resource utilization, and costs after LAI initiation and had fewer long-term care visits and home services than patients initiated on OAPs (22–24). A series of long-term study on the comparison OAPs with LAIs had conducted by Fernández-Miranda JJ, et al. to gain the evidences that the rates of discontinued treatment, hospital admissions, and suicide attempts were much higher in OAPs group than that in SGA-LAI group, thus SGA-LAIs should be considered more suitable for people with severe schizophrenia (25–29).

Despite the obvious advantages of PP1M, it has not been optimally used in clinical practice (30) and its application varies among different countries and regions. The usage rate of PP1M in Europe and America has been reported to be about 20%; however, it is lower in Asia (an average of 17.9%), and in the Chinese mainland, it has been reported to be only 0.66% (31–33). Factors that adversely affect the widespread use of LAIs include insufficient awareness regarding such medications in doctors and patients, patients’ fear of injections (34, 35), as well as access to these medications and health care policies within different regions (36, 37). Zhang Yan et al. reported that age, family per capita monthly income, dose of the third injection, and self-knowledge were the independent factors influencing the compliance of PP1M treatment in Chinese patients (38). Another important factor is that the expensive pharmacy patent fee may not match its slightly high clinical outcome (39).

This study aimed to obtain primary information regarding treatment patterns, healthcare resource utilization, and treatment costs including direct and total costs of patients with SZ one year before and after PP1M treatment, with an objective of providing comprehensive evidence in the real world for psychiatrists, patients, and healthcare decision-makers and providers. It would also benefit management care organizations, behavioral health organizations, and health policymakers and enable them to resolve the disease burden.

## 2 Methods

### 2.1 Subjects

This study was a retrospective, one-year mirror analysis in the real world. The data of study subjects came from the hospital information system of a Mental Health Units. The index date was determined to be the date of PP1M initiation, the pre-index phase was the one-year period of OAP use and the post-index phase was the PP1M phase for one year.

The inclusion criteria were as follows: 1) the age of the patient in year before the mirror point had to be 18–65 years old, 2) patients diagnosed with schizophrenia (ICD-10), and 3) patients who can be followed up for one year before and one year after using PP1M. According to the recommendations, the dosage of PP1M was as follows: “150 mg of PP1M to be injected on the first day of the treatment, 100 mg is to be injected again after a week; after the second dose, PP1M is to be injected once a month.” If the monthly dose is missed, patients can be administered the injection up to 7 days before or after the monthly time point. Therefore, the time after conversion to PP1M ended on the day of injection of the 13^th^ dose and corresponded with the time window allowed by the instruction. The exclusion criteria included: 1) pregnant or lactating women, 2) alcohol or drug abusers, 3) patients with severe comorbidities, 4) patients with organic brain disease, 5) mental retardation, 6) patients who could not be followed for least one year, 7) patients with ≤4 dosages/year of PP1M, and 8) patients who had received other LAIs including FGA-LAIs such as haloperidol decanoate injection and second generation antipsychotics (SGA)-LAIs such as RLAI within one year prior to recruitment. Ethics approval was obtained from the Medical Ethical Committee of Xiamen Xianyue Hospital (2020-KY-014). As the retrospective study was conducted using hospital information system records and the patients were not subjected to any intervention, informed consent was waived by the Medical Ethical Committee of Xiamen Xianyue Hospital.

### 2.2 Cost estimation

Owing to the limitation of data availability and operation, only direct expenses were included in this study. The direct medical expenses that were directly obtained from the hospital information system, mainly included research pharmacy expenses, accompanying pharmacy expenses, consultation expenses, physical examination expenses, hospitalization expenses, emergency expenses, and laboratory expenses.

Direct nonmedical expenses only included the transportation expenses incurred at each treatment. The final calculation of the transportation expenses was based on the frequency of hospitalization, emergency room visits, and outpatient visits recorded in the hospital information system, and was determined as “local” or “nonlocal” depending on the administrative area of patients’ address. Indirect and intangible costs were not included in the analysis. All costs have been expressed in 2023 RMB.

### 2.3 Statistical analysis

The software SPSS version 23.0 was used to perform the statistical analyses. Demographic characters including age and gender were described at baseline. Data means and standard deviations were used for continuous variables using the *T*-test, whereas classified variables were expressed in terms of counts and percentages. Paired *Wilcoxon* signed-rank tests were performed to compare differences in the cost between the pre/post-PP1M phases. Paired T-tests were used for comparing differences in times of hospitalization and inpatient days during the phases of pre/post-PP1M. Statistical differences were considered significant at *P* < 0.05.

## 3 Results

A total of 72 patients (defined as “group whole”) (43.06% female) with documented PP1M treatment were included in the analysis. The average age was (35.33 ± 12.33) years, of which 48 (66.67%) were 18–40 years of age. After switching to PP1M, the cost of PP1M accounted for 88.48% of the medication cost and 74.71% of the total treatment cost. Based on whether the patients had been hospitalized before using PP1M, subjects were divided into hospitalization group (defined as “group A”) and nonhospitalization group (defined as “group B”).

In all subjects, the use of health care resources including hospitalization and emergencies considerably decreased after conversion to PP1M (*P* < 0.001). Pharmacy costs increased by 222.57% (*P* < 0.001); however, the total cost of treatment in one year only increased by 16.35% (*P* < 0.001). The direct nonmedical costs also reduced by 26.58% (*P* = 0. 056), as depicted in **Table 1**.

**Table 1:** Treatment modalities, health care resource utilization, and burden for all subjects (n = 72).

Phases	Numbers of hospitalization	Days in hospital	Numbers of ER visits*	Pharmacy cost	Direct nonmedical cost	Total cost
pre-PP1M	1.10 ± 1.90	32.07 ± 55.64	1.96 ± 6.59	7492.31 ± 6102.90	2421.53 ± 1902.16	24978.94 ± 23127.85
post-PP1M	0.08 ± 0.44	2.36 ± 12.05	0.21 ± 1.03	24542.42 ± 7293.08	1777.78 ± 1000.49	29063.81 ± 8936.28
Difference	−92.41%	−92.64%	−89.36%	222.57%	−26.58%	16.35%
*P*	<0.001	<0.001	<0.001	<0.001	0.056	<0.001

*ER, emergency room; PP1M, paliperidone palmitate once-monthly.

Only two patients in group A were rehospitalized after conversion to PP1M, and both of them were hospitalized in OAPs phase. After switching to PP1M, the usage of healthcare resources, including hospital and emergency room visits, significantly decreased (*P* < 0.001). In group A, The hospitalization cost in OAPs phase accounted for 82.04% of the total cost, whereas the PP1M cost accounted for 91.10% of the total pharmacy cost and 77.04% of the total cost after changing the treatment modality. Direct nonmedical costs and total costs decreased by 16.92% (*P* = 0.793) and 37.67% (*P* < 0.001), respectively. By contrast, the outpatient registration fee and pharmacy cost increased by 59.72% (*P* = 0. 003) and 185.21% (*P* < 0.001), respectively (**Table 2**).

**Table 2:** Treatment modalities, health care resource utilization, and burden of schizophrenia with ≥1 hospitalization pre-PP1M (n = 25).

Phases	Times of hospitalization	Days in hospital	Times of ER visits*	Pharmacy cost	Direct nonmedical cost	Total cost
pre-PP1M	3.12 ± 2.03	91.16 ± 59.58	1.64 ± 1.89	8429.14 ± 7590.26	1856.00 ± 1229.78	45608.74 ± 26423.57
post-PP1M	0.16 ± 0.62	4.4 ± 16.61	0.16 ± 1.89	24040.74 ± 6767.63	1542.00 ± 601.16	28428.3 ± 7952.45
Difference	−94.87%	−95.17%	−90.24%	185.21%	−16.92%	−37.67%
*P*	<0.001	<0.001	<0.001	<0.001	0.793	<0.001

*ER, emergency room; PP1M, paliperidone palmitate once-monthly.

No hospitalization case was found in group B after being converted to PP1M. In the PP1M phase, the number of emergency and outpatient services were observed to have reduced by 88.66% (*P* < 0.001) and 30% (*P* = 0.012), respectively. In addition to pharmacy and registration expenses, the direct medical expenses and direct nonmedical expenses reduced by 57.07% (*P* = 0.997) and 30.17% (*P* = 0.055), respectively. By contrast, registration fee, pharmacy cost, and total treatment cost increased by 6.18% (*P* = 0.0892), 260.15% (*P* < 0.001), and 117.56% (*P* < 0.001), respectively, as shown in **Table 3**. In group B, the cost of PP1M accounted for 87.12% of the total pharmacy cost (**Table 4**) and 73.52% of the total treatment cost.

**Table 3:** Treatment modalities, health care resource utilization, and burden of schizophrenia with nonhospitalization pre-PP1M (n = 47).

Phases	Numbers of ER visits*	Numbers of outpatient visits	Examination cost	Pharmacy cost	Direct nonmedical cost	Total cost
pre-PP1M	2.06 ± 8.07	31.28 ± 19.28	2911.83 ± 6555.05	6888.59 ± 5226.05	2725.53 ± 2127.00	13514.53 ± 10269.21
post-PP1M	0.23 ± 1.20	21.81 ± 9.76	1250.01 ± 3226.93	24809.28 ± 7615.15	1903.19 ± 1144.62	29401.85 ± 9482.92
Difference	−88.66%	−30.27%	−57.07%	260.15%	−30.17%	117.56%
***P***	<0.001	0.012	0.997	<0.001	0.055	<0.001

*ER, emergency room; PP1M, paliperidone palmitate once-monthly.

**Table 4:** Difference of the cost between Groups A and B before and after using PP1M.

Group	pre-PP1M			post-PP1M				
	Pharmacy cost	Direct nonmedical cost	Total cost	Pharmacy cost	Direct nonmedical cost	Total cost	PP1M dosages	PP1M cost
Group A	8429.14 ± 7590.26	1856.00 ± 1229.78	45608.74 ± 26423.57	24040.74 ± 6767.63	1542.00 ± 601.16	28428.30 ± 7952.45	10.96 ± 2.96	21900.1 ± 6595.02
Group B	6888.59 ± 5226.05	2725.53 ± 2127.00	13514.53 ± 10269.21	24809.28 ± 7615.15	1903.19 ± 1144.62	29401.85 ± 9482.92	10.60 ± 3.25	21614.94 ± 8078.38
t	−1.014	1.88	−7.379	70	1.47	0.438	−0.688	−0.152
*P*	0.314	0.064	<0.001	0.673	0.146	0.663	0.494	0.88

PP1M, paliperidone palmitate once-monthly.

For understanding the difference in cost between hospitalized and nonhospitalized patients during the OAPs phase, a further comparative analysis was conducted addressing the usage of healthcare resources before and after using PP1M. The results demonstrated no statistical difference in pharmacy cost, direct nonmedical cost, dosages, and cost of PP1M between hospitalized and nonhospitalized patients within pre/post-PP1M phase (*P* > 0.05). In the pre-PP1M phase, the total cost of the hospitalized group was 237.48% higher than that of the nonhospitalized group (*P* < 0.001); however, no statistical difference was observed in the post-PP1M phase (*P* < 0.663) indicating that the proportion of nonpharmacy cost during hospitalization was high (**Table 4**).

## 4 Discussion

This mirror image study demonstrated that after switching from OAPs to PP1M, the usage of healthcare resources, including hospitalization and emergency, and direct pharmacy expenses, such as times of hospitalizations, days spent in the hospital, expenses associated with hospitalizations, times and of emergency room visits, and the associated expenses considerably decreased, accompanied by a decrease in direct nonmedical expenses and a considerable increase in pharmacy expenses with PP1M contributing to more than 85% of the pharmacy expenses. For those with ≥1 hospitalizations, although the pharmacy cost considerably increased after conversion to PP1M, the total one-year treatment cost reduced by 37.67%, resulting in a considerable reduction in the direct cost. However, an increase in cost by 117.56% among those without hospitalizations indicated that nonpharmacy direct medical expenses accounted for a large proportion during hospitalization, and after conversion to PP1M, the financial structure exhibited an obvious change.

This result was consistent with other domestic reports. Wu et al. (40) reported that SZ-related costs in the hospitalized group were 11 times greater than that of the nonhospitalized group ($2,772 versus $231, *P* < 0.001). In terms of the subgroups, nonmedication treatment costs accounted for 48.1% of the expenses for the hospitalized group. And the major expenses for the nonhospitalized group included medical costs (95.6%). Another previously conducted retrospective analysis of the cost structure of hospitalized patients with SZ from 2014 to 2019 demonstrated that pharmacy costs only accounted for 14.07% of the direct medical expenses during hospitalization, and more than 80% were other direct medical expenses, including treatment expenses, bed and nursing fees, examination costs and consultation costs (41).

The previous study and this study demonstrated that PP1M was associated with outstanding advantages in conserving medical resources and reducing costs. Compared with the previous period in which oral antipsychotics were used, after treatment with PP1M for 12 months, average hospitalization days decreased by 51.64 days in the Chinese population, and the total hospitalization expenses decreased by 1,991 US dollars, which was equivalent to 6,698 dollars and 6,716 dollars in Korea and Malaysia (42). According to a real-world observational study, patients treated with LAIs had lower monthly inpatient and emergency room visits costs but higher monthly medication costs over the 12-month postindex period than the oral cohort. Patients prescribed PP1M were associated with lower adjusted all-cause medical costs and shorter lengths of hospitalizations that patients prescribed OAPs. Fewer hospitalizations and shorter lengths of hospitalizations during treatment with PP1M also contributed to lower monthly hospitalization expenses (43, 44). This study found that after converting to PP1M, the total treatment cost increased by 1.1 times, among which the pharmacy cost increased by 2.6 times for those patients who were not hospitalized during the OAPs phase, and were also defined as “completely treatment compliant.” The increase in the ratio of pharmacy cost was far greater than increase in total treatment costs, which might indicate that PP1M was associated with lesser obvious advantages for patients who were compliantly taking OAPs in a short-term period. By contrast, PP1M would be a good therapeutic schedule for patients with one or more than one hospitalizations. Most evidence-based clinical guidelines have recommended the use of LAIs for patients with a history of poor adherence and relapse (45, 46). However, the costs of PP1M accounted for more than 70% of the total pharmacy cost and total treatment cost, regardless of whether the patient was hospitalized in the OAPs phase. In addition, the high total treatment cost was mainly attributed to the expensive product.

The direct nonmedical costs in this study only included transportation expenses, while accommodation expenses, nutritional food expenses, etc., were excluded, which may also affect the conclusion. Previously procured data have indicated that direct nonhealthcare excess costs, including living cost offsets, were estimated to account for 6%–12% of the economic burden in patients with SZ (47–49), and we believe that more than a quarter saving (26.58%) bears a positive economic and sociological significance for patients and society.

Herein, after switching to PP1M, only two patients were hospitalized in all subjects group and both were hospitalized during the OAP phase. In addition, the notable reduction in the number of emergency room visits further verified better medication compliance and clinical efficacy of PP1M, and the results were consistent with several previous reports (50, 51). A previous retrospective longitudinal study had demonstrated that after using PP1M, the annual average pharmacy cost and outpatient cost increased by 3,417 and 2,527 dollars, respectively; however, the annual average hospitalization cost decreased by 14,456 dollars. Thus, the reduction in the number of hospitalizations also reduced the hospitalization cost (52). A previous systematic review had demonstrated that compared with OAPs, the average all-cause hospitalization rate and hospitalization days were considerably reduced with LAIs, resulting in a substantial reduction in the average all-cause hospitalization and schizophrenia-related hospitalization expenses (53). Results from studies compared different paliperidone formulations demonstrated that apart from somewhat improvement in treatment adherence, effectiveness, and tolerability, patients treated with PP1M showed more satisfaction with PP3M. a novel formulation of paliperidone three monthly LAI, and longer-duration formulations should be a better choice (54, 55).

The results of this study indicated that, irrespective of presence or absence of hospitalized in the OAPs phase, few direct medical expenses including inspection, physical examination and testing expenses, had decreased by more than 50% after switching to PP1M. The reasons could be attributed to good patient compliance with PP1M, longer half-life (as long as 25–49 days) of PP1M in plasma, stable serum drug concentration, lesser fluctuation of patients’ condition, and lesser related examinations and testings were required (56, 57). However, various factors including impaired insight into illness, psychopathology, substance use disorder, issues associated with treatment, stigma, the efficacy and tolerability of antipsychotics, and socioeconomic status, poor compliance was commonly associated with treatment with OAPs (58), which caused the fluctuation of illness, and eventually led to the need for more examination and testing.

Unexpectedly, no statistical difference was observed in outpatient registration fees before and after using PP1M. This could be attributed to the high price of PP1M, which was usually managed as valuable medication. Also, according to local health administration policies (59), PP1M can be prescribed only by doctors with high seniority qualifications (whose consultation fee are also higher), thereby resulting in higher registration fees.

The results of the study should be treated cautiously because of the limitations of the study design. First, cases of loss to follow-up and missed data were unavoidable in this retrospective analysis owing to factors such as course of disease and data of clinical efficacy. Second, factors such as replacement of the information system, or patients seeking medical service in different institutions, where data was not shared, resulted in a smaller sample size of patients who met the criteria. Third, the results might not represent the complete Chinese population as most of the subjects included were permanent residents. Fourth, this study failed to distinguish the cost categories of patients, such as employee medical insurance or resident medical insurance or self-funded, which could also affect the patients’ choice of healthcare resources, and costs. Fifth, it would be slightly short of one-year research for the refractory and needed long-term treatment diseases, resulting in insufficient economic advantages of PP1M. Finally, the indirect costs owing to the loss of productivity and the care of family members, which could account for 50.5%–73.4% of the economic burden of SZ (49, 60, 61) has not been included in this study, so the aggregate expansion of the disease was seriously underestimated.

In the follow-up research, we will coordinate with more departments and regions, include more samples, extend the follow-up time appropriately, or adopt prospective observational research methods to collect accurate and comprehensive data on SZ treatment, such as clinical and cost data, so as to provide more reliable evidence for management decision-makers and ultimately benefit more patients.

## 5 Conclusions

This study estimated the usage of healthcare resources and direct medical and direct nonmedical expenses of SZ patients before and after switching from OAPs to PP1M. The results demonstrated that after switching to PP1M, the number and lengths of hospitalization days and the number of outpatient and emergency room visits considerably decreased, thereby affecting the accompanying direct medical and direct nonmedical expenses, which reduced repeated medical services and reduced economic burden. In addition to its clinical benefits, PP1M may also reduce the financial burden of the difficult-to-treat disease by reducing relapses. Nonpharmacy expenses during hospitalization accounted for a large proportion of the total hospitalization expenses in patients with hospitalization experience, and the reductions in health resources and total expenses were considerable after switching to PP1M, which indicated that these patients might benefit more by early initiation of PP1M. The cost of PP1M was the main contributor to the pharmacy cost and total cost. The high acquisition cost of PP1M could probably be an obstacle toward its widespread use, and multifaceted effort is required.

## Data Availability

The original contributions presented in the study are included in the article/Supplementary Material. Further inquiries can be directed to the corresponding author.
